# Sodium tanshinone IIA sulfonate protects vascular relaxation in ApoE-knockout mice by inhibiting the SYK-NLRP3 inflammasome-MMP2/9 pathway

**DOI:** 10.1186/s12872-024-03990-0

**Published:** 2024-07-12

**Authors:** Hai-Hua Liu, Wei Wei, Fei-Fei Wu, Lu Cao, Bing-Jie Yang, Jia-Ning Fu, Jing-Xia Li, Xin-Yue Liang, Hao-Yu Dong, Yan-Yan Heng, Peng-Fei Zhang

**Affiliations:** 1 Department of Endocrinology, Heping Hospital Affiliated to Changzhi Medical College, No.110, Yan’an South Road, Changzhi, 046000 Shanxi China; 2 Department of Pharmacology, Changzhi Medical College, No.161, Jiefang East Street, Changzhi, 046000 Shanxi China; 3 Department of Clinical Center Laboratory, Heping Hospital Affiliated to Changzhi Medical College, No.110, Yan’an South Road, Changzhi, 046000 Shanxi China; 4 Department of Stomatology, Changzhi Medical College, No.161, Jiefang East Street, Changzhi, 046000 Shanxi China; 5 Department of Anesthesia, Changzhi Medical College, No.161, Jiefang East Street, Changzhi, 046000 Shanxi China; 6 Department of Medical Imageology, Changzhi Medical College, No.161, Jiefang East Street, Changzhi, 046000 Shanxi China; 7 Department of Nephrology Heping Hospital, Changzhi Medical College, No.110, Yanan Road South, Changzhi, 046000 Shanxi China

**Keywords:** Hyperlipidemia, Vascular relaxation, NLRP3 inflammasome, MMP2/9, Sodium tanshinone IIA sulfonate

## Abstract

**Background:**

Hyperlipidemia damages vascular wall and serves as a foundation for diseases such as atherosclerosis, hypertension and stiffness. The NOD-like receptor family pyrin domain-containing 3 (NLRP3) inflammasome is implicated in vascular dysfunction associated with hyperlipidemia-induced vascular injury. Sodium tanshinone IIA sulfonate (STS), a well-established cardiovascular protective drug with recognized anti-inflammatory, antioxidant, and vasodilatory properties, is yet to be thoroughly investigated for its impact on vascular relaxant imbalance induced by hyperlipidemia.

**Methods:**

In this study, we treated ApoE-knockout (ApoE-/-) mouse with STS and assessed the activation of the NLRP3 inflammasome, expression of MMP2/9, integrity of elastic fibers, and vascular constriction and relaxation.

**Results:**

Our findings reveal that STS intervention effectively preserves elastic fibers, significantly restores aortic relaxation function in ApoE-/- mice, and reduces their excessive constriction. Furthermore, STS inhibits the phosphorylation of spleen tyrosine kinase (SYK), suppresses NLRP3 inflammasome activation, and reduces MMP2/9 expression.

**Conclusions:**

These results demonstrate that STS protects vascular relaxation against hyperlipidemia-induced damage through modulation of the SYK-NLRP3 inflammasome-MMP2/9 pathway. This research provides novel insights into the mechanisms underlying vascular relaxation impairment in a hyperlipidemic environment and uncovers a unique mechanism by which STS preserves vascular relaxation, offering valuable foundational research evidence for its clinical application in promoting vascular health.

**Supplementary Information:**

The online version contains supplementary material available at 10.1186/s12872-024-03990-0.

## Background

The hyperlipidemic environment impairs vascular wall function, potentially serving as an inducing factor for vascular complications such as hypertension, atherosclerosis, and vascular wall stiffening [1–3]. Factors such as high cholesterol and triglycerides deposit beneath the endothelial cells (ECs) layer, inducing oxidative stress and inflammatory reactions. This leads to the migration and extensive proliferation of vascular smooth muscle cells (VSMCs) into the subendothelial layer, generating neointima and encroaching upon the vascular lumen space, ultimately forming atherosclerotic lesions [4, 5]. Simultaneously, during the prolonged pathological induction of hyperlipidemia, vascular remodeling, loss of vascular wall elasticity, fibrosis, and calcification can occur [6–8]. Therefore, hyperlipidemia induces vascular damage with severe prognostic implications. Recent studies have revealed that the activation of the NOD-like receptor family pyrin domain-containing 3 (NLRP3) inflammasome plays a pivotal role in vascular damage induced by hyperlipidemia.

The NLRP3 inflammasome is a crucial component of the innate immune system and has been demonstrated to be associated with numerous significant human diseases [9]. The activation of the NLRP3 inflammasome is widely acknowledged to instigate dysfunction in VSMCs, encompassing pyroptosis, phenotypic switching, calcification, proliferation, and migration [10–13]. Therefore, NLRP3 inflammasome plays a crucial role in hyperlipidemia-induced vascular damage, particularly in the progression of atherosclerosis and vascular wall stiffness. Furthermore, NLRP3 inflammasome activation is associated with matrix metalloproteinase 9 (MMP9)-dependent impairment of vascular elastic fibers [14]. However, in hyperlipidemia, the role and mechanisms by which the NLRP3 inflammasome mediates impairment of vascular elastic function remain unclear. The current study provides partial insights into the potential mechanisms through which hyperlipidemia activates the NLRP3 inflammasome, contributing to the impairment of vascular elastic function.

Sodium Tanshinone IIA Sulfonate (STS) is a water-soluble monomeric chemical compound derived from the traditional Chinese herb *Salvia miltiorrhiza*, commonly known as Danshen [15]. It has been used for many years in clinical treatment of cardiovascular diseases. STS inhibits proliferation and migration of VSMC, and its mechanisms may be associated with the inhibition of inflammatory pathways [16, 17]. However, further investigation is needed to determine whether STS protects vascular elastic function in the context of hyperlipidemia.

This study elucidates that STS protects vascular relaxant function by inhibiting the activation of the NLRP3 inflammasome and reducing MMP2/9 levels. The findings unveil a novel mechanism underlying the impairment of vascular relaxant function in the context of hyperlipidemia.

## Methods

### Animal

ApoE-knockout (ApoE-/-) and wild type (WT, control, Ctrl) mice (Male, 6 weeks old, weight 20–25 g) were purchased from SPF-animals (Beijing, China). All mice were housed in a facility (22℃, 45% humidity, 12 h/12 h light/dark cycle), where they ingested water and food freely. ApoE-/- mice were fed with western diet (No.10,142, D12108C, 40% high-fat, 1.25% cholesterol, Sinodiets, Siping, China) from 7 weeks old to 12 weeks old, then the mice were sacrificed (Carbon dioxide asphyxiation) and the aortas were collected and fixed by 4% paraformaldehyde (PH0427, PHYGENE, Fuzhou, China) and embedded in paraffin or cut into a 3 mm width segments to detect the tension. The plasma total cholesterol (TC) and triglyceride (TG) were measured using commercial kits (A111-1, Nanjing Jiancheng Bioengineering Institute, Nanjing, China; BC0625, Solarbio, Beijing, China).

### Verhoeff Van Gieson (VVG) staining

The elastic fiber in the aorta wall (adventitia removed) was assessed using a commercial assay kit (BP-DL201, Sbjbio, Nanjing, China). We selected tissue from the ascending aorta and the proximal part of the aortic arch (areas experiencing stronger blood flow impact) for paraffin embedding. After dewaxing paraffin sections, Verhoeff staining solution was applied to cover the aortic tissue sections for approximately 30 min. After rinsing away the stain with running water, Verhoeff differentiation solution was applied dropwise and observed under a microscope until elastic fibers became clearly visible. Following thorough rinsing with running water, Van Gieson staining solution was applied for 1 min. The sections were then dehydrated in ethanol, cleared in xylene, and mounted with neutral resin. Images were captured at 400× magnification, and the proportion of elastic fibers in the vascular wall was quantified using ImageJ software.

### Immunohistochemistry (IHC)

The aortas (adventitia removed) were extracted from the mice treated with STS (intravenously injection, i.v., 10 mg/kg/day, S107694, Aladdin, Shanghai, China), SB-3CT (intraperitoneal injection, i.p., 50 mg/kg, every other day, HY-12,354, MCE, Shanghai, China), a small-molecule MMP2/MMP9 inhibitor [18], MCC950 (10 mg/kg, twice weekly i.p., HY-12,815 A, MCE, Shanghai, China), a selective NLRP3 inhibitor [19], R406 (5 mg/kg/d, orally, HY-12,067, MCE, Shanghai, China), a Spleen Tyrosine Kinase (SYK) inhibitor [20]. After thorough dewaxing of the sections, they were immersed in sodium citrate antigen retrieval solution (PH0422, phygene, Fuzhou, China) and heated to boiling, followed by cooling to room temperature. Subsequently, they were treated with 3% hydrogen peroxide (H_2_O_2_, PH1884, Phygene, Fuzhou, China) for 10 min, followed by blocking with 5% Bovine Serum Albumin (BSA, B265993, Aladdin, Shanghai, China) for 1 h. The sections were incubated with the primary antibodies, rabbit anti-MMP2 (1:100, CY7164, Abways, Shanghai, China), rabbit anti-MMP9 (1:100, WL03096, Wanleibio, Shenyang, China), rabbit anti-pSyk-try525 (1:100, AY8126, Abways, Shanghai, China), rabbit anti-NLRP3 (1:100, Abways, CY5651, Shanghai, China) overnight at 4 °C, followed by the second antibody Goat anti-Rabbit IgG (1:200, AB0101, Abways, Shanghai, China) incubation for 1.5 h at regular temperature. DAB (3,3′-Diaminobenzidine) (PH0728, PHYGENE, Fuzhou, China) was used as a chromogen. The percentage of DAB positive area was measured using Image J software. The non-specific binding control is presented in Supplementary Fig. 2.

### Vascular tension recording

The contraction and relaxation functions of aortas was detected by tension detection system (BL-420 S, TaiMeng, Chengdu, China) as described previously [13, 21]. Mice were anaesthetized and aortas was quickly removed and immersed into Krebs Henseleit solution (mM) (KH, pH 7.4, 119.0 NaCl, 25.0 NaHCO_3_, 11.1 Glucose, 2.4 CaCl_2_, 4.7 KCl, 1.2 KH_2_PO_4_, 1.2 MgSO_4_,0.024 Na_2_EDTA). Aortas were carefully dissected into a transparent tube, and then cut into vascular rings with a width of approximately 3 mm. The endothelium of aortas was removed using a flexible wire (0.38 mm diameter). The vascular rings were then suspended in a water-jacketed tissue bath and the tension tested. KH solution was maintained at 37 °C and the mixed gas containing 95% O_2_, and 5% CO_2_ was continuously bubbled through the bath. When the tension of the rings stabilized at the basal level, the rings were assessed for contraction and relaxation function using phenylephrine (Phe, 1 × 10^− 9^-10^− 5^ M, S161304, Aladdin, Shanghai, China) and sodium nitroprusside (SNP, 1 × 10^− 9^-10^− 5^ M, S305727, Aladdin, Shanghai, China), the record of relaxation induced by Phe (1 × 10^− 4^ M), SNP (1 × 10^− 4^ M) in the Ctrl group was set as 100% response to the Phe or SNP.

### Immunofluorescence (IF) analysis

After de-paraffinization, antigen retrieval (PH0422, Phygene, Fuzhou, China) vascular sections were treated with 3% hydrogen peroxide (H_2_O_2_, PH1884, Phygene, Fuzhou, China) and blocked with 5% BSA (B265993, Aladdin, Shanghai, China). The sections were incubated with the primary antibody mouse anti-NLRP3 (1:100, 68102-1-Ig, Proteintech, Wuhan, China), rabbit anti-TMS1 (ASC, 1:100, CY5689, Abways, Shanghai, China) overnight at 4°C, and the second fluorescent antibody Goat Anti-Mouse IgG (H + L) Alexa Fluor 594 (1:200, AB0151, Abways, Shanghai, China), Goat Anti-Rabbit IgG (H + L) Alexa Fluor 488 (1:200, AB0142, Abways, Shanghai, China). 4’,6-diamidino-2-phenylindole (DAPI) (C1006, Beyotime, Shanghai. China) labeled nuclei. Images were photographed by a fluorescent microscope (FRD-6 C, Cossim, Beijing, China). The colocalization between NLRP3 and ASC was quantified using the Pearson Correlation Coefficient, calculated with ImageJ software (NIH, Littleton, CO, USA).

### Detection of active Caspase-1, IL-1β, and IL-18 levels

The Caspase-1 activity was determined using a commercial Caspase-1 Activity Assay Kit (C1101, Beyotime, Shanghai, China) as previously described [22]. Arterial homogenate was centrifuged at 15,000 rpm/min for 15 min at 4 °C. The supernatants were collected and qualified by a BCA assay kit (P0010, Beyotime, Shanghai, China). Caspase-1 activity in an equal amount of protein, approximately 200 µg, was determined immediately. Ac-YVAD-pNA was added to the supernatant and incubated for 60–120 min at 37 °C. When the solution exhibited a distinct yellow color, the samples were measured using a microplate reader (Thermo Fisher Scientific, USA) at 405 nm. Active levels of IL-1β and IL-18 in aorta (endothelium and adventitia removed) homogenates were determined using commercial ELISA kits (SEKM-0002, SEKM-0019, Solarbio, Beijing, China), according to kits specification, and the optical density (OD) was measured at 450 nm.

### Western blot (WB)

WB analysis was performed as described previously [13]. Briefly, aortas were homogenized after removal of the intima and adventitia, aorta protein samples were extracted by using Mammalian active protein extraction reagent (Beyotime, P0013M, Shanghai, China). Protein concentration was quantified by using a BCA Protein Assay Kit (Beyotime, P0010S, Shanghai, China). According to the quantification, protein samples were adjusted to equal with 5×loading buffer (Beyotime, P0015, Shanghai, China), and samples were boiled for 5 min at 95 °C. Equal amounts of cell lysate protein were resolved by SDS-PAGE using 4% (w/v) stacking and 10% (w/v) separating polyacrylamide gels. The whole piece of separated gel was cut according to the molecular weight of the target protein, then the cut-gels were transferred to 0.45 μm polyvinylidene difluoride membrane (PVDF, Millipore, USA), which were then blocked for 1.5 h in 5% (w/v) nonfat milk diluted in Tris-buffered saline (TBS, 100 mM Tris–HCL, pH 7.4) with 0.2% (v/v) Tween-20, and incubated with the primary antibodies rabbit anti-pSYK-try525 (2710, 1:1000, cell signaling technology Shanghai, China) and rabbit anti-SYK (CY3461, 1:1000, Abways Technology, Inc., Shanghai, China) overnight at 4 °C. After being washed for three times, the membranes were incubated with goat anti-rabbit IgG (1:5000, Abways Technology, Inc., Shanghai, China) for 1.5 h. The blot was detected by an automatic chemiluminescence/fluorescence image analysis system (5200 Multi, Tanon, Shanghai, China) with electrochemiluminescence (ECL, P0015, Beyotime, Shanghai, China Shanghai, China). Densitometric analysis of the images was performed with Image J software (NIH, Littleton, CO, USA). The cropped WB bands shown in Fig. 2 of the main text are presented in full length in Supplemental Fig. 3.

### Detection of left ventricular blood pressure (LVBP)

LVBP detection was performed as previously described [23]. Briefly, mice were induced with 3% isoflurane inhalation (R540, RWD, Shenzhen, China) until they completely lost consciousness and pain sensation, then maintained with 2% isoflurane. A 0.33 mm diameter catheter was introduced into the left ventricular from the right common carotid artery. After the heart rate of mice stabilized, the systolic and diastolic blood pressures were recorded using a biological signal acquisition system (BL-420 S, TaiMeng, Chengdu, China) connected with a pressure transducer (YP200, Zhenhua Teaching Instrument Company, Xinxiang, China).

### Statistics

Statistical analysis was performed with Graphpad PRISM 9.0 software. Data are presented as means ± SD. Significant differences between and within multiple groups were examined using ANOVA for repeated measures, followed by Tukey’s multiple comparisons test. The Independent-Samples t-test was used to detect significant differences between two groups. *P* < 0.05 was considered statistically significant.

## Results

### STS preserves the elastic fibers and vascular relaxation while reducing the expression of MMP2/9 in vascular walls of APOE-/- mice

**Fig. 1:**
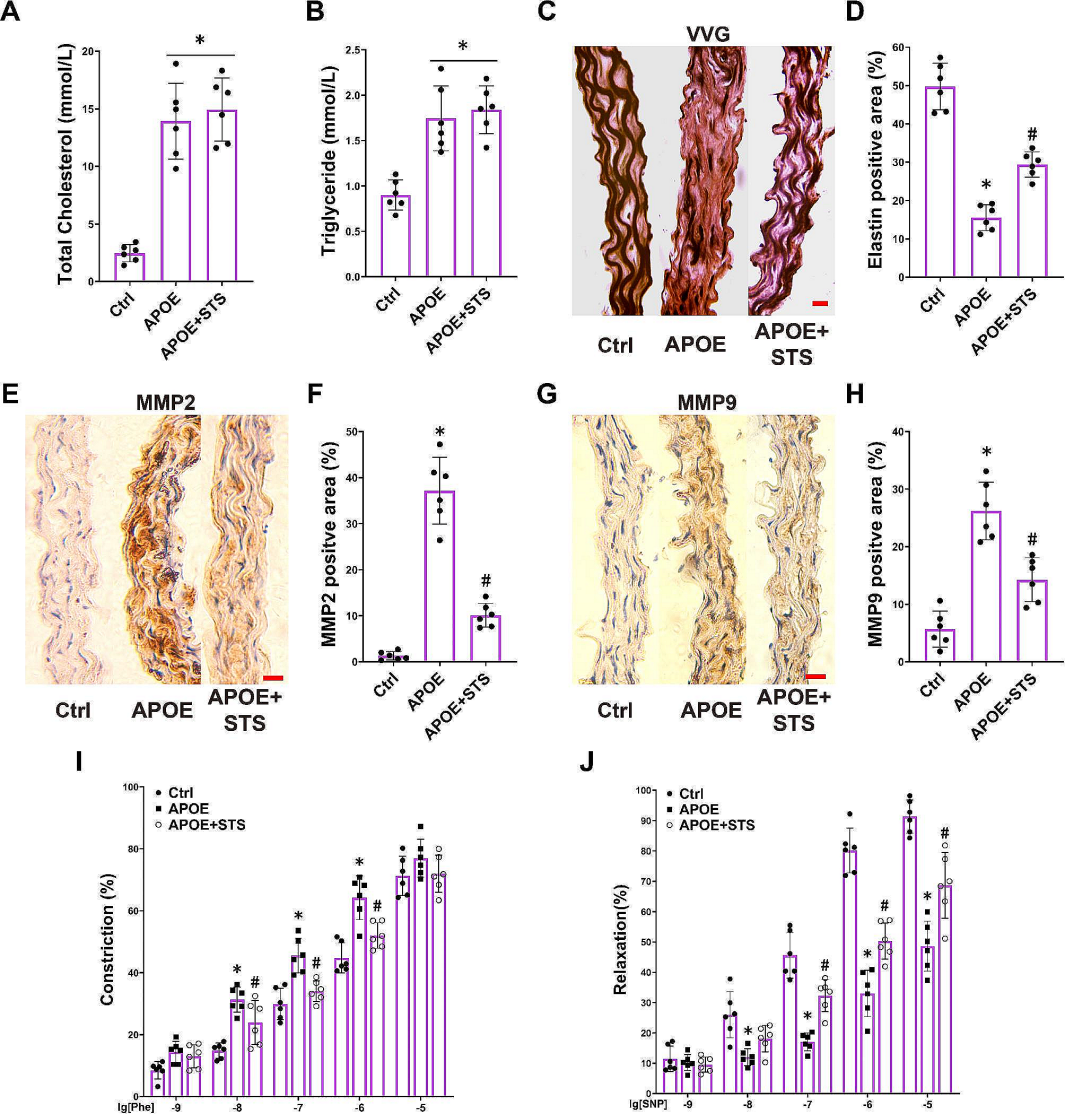
STS preserves the elastic fibers and vascular relaxation while reducing the expression of MMP2/9 in vascular walls of APOE-/- mice. Ctrl: wild-type mice without any intervention; APOE: APOE-/- mice that did not undergo any intervention; APOE + STS: APOE-/- mice that received intravenous injection of STS. (**A-B**): The summarized data show the levels of total cholesterol (TC) and triglycerides (TG) in plasma (Data represent means ± SD, one-way ANOVA); (**C-D**): Representative VVG staining images (400×) and summarized data show the elastic fiber positive area in aortas (Data represent means ± SD, one-way ANOVA); (**E-H**): Representative IHC images (400×) and summarized data show the MMP2 and MMP9 positive area in aortas (Data represent means ± SD, one-way ANOVA); (I-J): The summarized data of vascular response to phenylephrine (Phe, 1 × 10^− 9^-10^− 5^ M, vascular constriction) and sodium nitroprusside (SNP, 1 × 10^− 9^-10^− 5^ M, vascular relaxation) was determined in aorta rings (Data represent means ± SD, two-way ANOVA). *P < 0.05 vs. Control (Ctrl); #*P* < 0.05 vs. APOE-/- (*n* = 6, scale bar = 20 μm)

We first assessed the lipid levels in ApoE-/- mice fed a high-fat diet. We observed that the plasma concentrations of total cholesterol (TC) and triglycerides (TG) in ApoE-/- mice were approximately 6 times and 1.7 times higher, respectively, compared to the control group mice (Fig. 1A and B). STS intervention did not show a significant impact on the TC and TG levels (Fig. 1A and B).

After administration of STS, we evaluated the integrity of elastic fiber, MMP2/9 expression, and vascular contractile and relaxant functions in the aortas of ApoE-/- mice. We observed disordered and fragmented elastic fibers in ApoE-/- mice, along with a noticeable decrease in elastic fiber area (Fig. 1C-D), accompanied by an upregulation of MMP2 and MMP9 expression (Fig. 1E-H); STS intervention preserved the integrity of elastic fibers, suppressed MMP2 and MMP9 expression in the aorta of ApoE-/- mice (Fig. 1C-H).

**Fig. 2:**
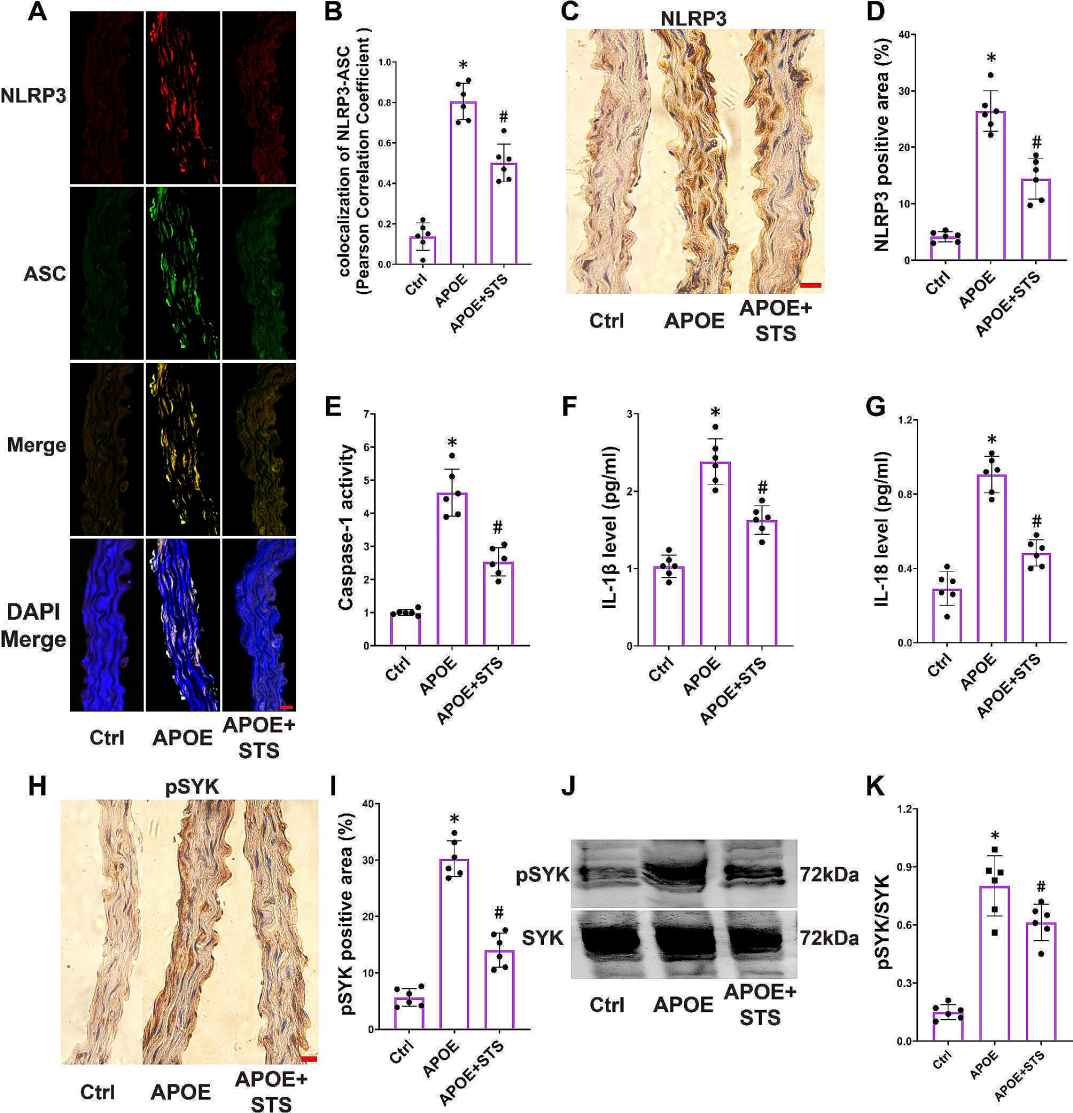
STS suppresses the activation of the NLRP3 inflammasome in the vascular wall of ApoE-/- mice. (**A-B**): Representative immunofluorescence images of NLRP3 and ASC co-localization (400×) in aortas and the summarized data show the Pearson correlation coefficient of NLRP3 and ASC; (**C-D**): Representative IHC images (400×) and summarized data show the NLRP3 positive area in aortas; (**E-G**): The summarized data show the activity of caspase-1 and the levels of IL-1β and IL-18 in aorta homogenate; (**H-I**): Representative IHC images (400×) and summarized data show the phosphorylation of SYK positive area in aortas; (**J-K**): Representative cropped WB image and summarized data showing pSYK/SYK, the full-length blots are presented in Supplemental Fig. 3. *P < 0.05 vs. Control (Ctrl); #*P* < 0.05 vs. APOE-/- (*n* = 6, scale bar = 20 μm). Data represent means ± SD, one-way ANOVA

Compared to the aortic rings from Ctrl (WT mice), the aortic rings from APOE-/- mice exhibit significantly stronger responses to 10^− 8^, 10^− 7^, and 10^− 6^ concentrations of Phe-induced constriction; at a 10^− 5^ concentration of Phe-induced constriction, the constriction strength of the aortic segments from APOE-/- mice no longer shows a significant difference compared to those from Ctrl (Fig. 1I). This indicate that, compared to Ctrl, the aortas of APOE-/- mice are more sensitive to contractile induction, exhibiting stronger constriction responses at weaker contractile inductions, while the response to stronger contractile induction remains unchanged. Importantly, the aortic rings from APOE-/- mice exhibited significantly reduced relaxation responses to SNP induction at concentrations ranging from 10^− 8^ to 10^− 5^ compared to those from Ctrl (Fig. 1J). This indicate that the aortas of APOE-/- mice have impaired relaxation function compared to those of Ctrl. STS intervention significantly mitigated the excessive constriction of aortic segments from APOE-/- mice induced by 10^− 8^, 10^− 7^, and 10^− 6^ concentrations of Phe, as well as significantly improved the reduced relaxation of aortic segments from APOE-/- mice induced by 10^− 7^, 10^− 6^, and 10^− 5^ concentrations of SNP (Fig. 1I and J). This indicate that STS protects the relaxation function of aorta in APOE-/- mice with hyperlipidemia.

Furthermore, through left ventricular blood pressure (LVBP) measurements, we found that APOE-/- mice did not exhibit a significant increase in left ventricular systolic pressure (supplemental Fig. 1A), but they did show a significant elevation in left ventricular diastolic pressure (supplemental Fig. 1B). STS intervention was able to suppress the elevated left ventricular diastolic pressure in APOE-/- mice (supplemental Fig. 1B). These results may suggest that STS intervention could partially alleviate the increased afterload in the left ventricle of APOE-/- mice, which could be associated with an improvement in vascular relaxation function.

These results demonstrate that STS intervention inhibits the expression of MMP2/9, preserves elastin fibers, protecting against hyperlipidemia-damaged vascular relaxation.

### STS suppresses the activation of the NLRP3 inflammasome in the vascular wall of ApoE-/- mice

Research indicates a significant downregulation of elastin level in the vascular wall during NLRP3 inflammasome activation [24]. We hypothesize that STS-mediated protection of elastic fibers alleviates vascular elasticity in ApoE-/- mice, potentially by inhibiting the activation of the NLRP3 inflammasome.

We observed enhanced colocalization of NLRP3 and ASC proteins in the vascular wall of ApoE-/- mice (Fig. 2A and B), upregulation of NLRP3 expression (Fig. 2C and D), and increased activity of caspase-1 (the core product of NLRP3 inflammasome activation, also known as IL-1β-converting enzyme) (Fig. 2E), the production of active IL-1β and IL-18 (Fig. 2F and G). Concurrently, we noted elevated positive phosphorylation of SYK at tyrosine 525 by IHC (Fig. 2H and I) and the ratio of pSYK/SYK by WB, (Fig. 2J and K). STS intervention suppressed these changes (Fig. 2A-K).

These findings indicate that STS inhibits vascular NLRP3 inflammasome activation induced by a hyperlipidemic environment, and this mechanism may be associated with the inhibition of SYK phosphorylation.

### STS protects elastic fibers and vascular relaxation by inhibiting MMP2/9 in the vascular wall of ApoE-/- mice

**Fig. 3:**
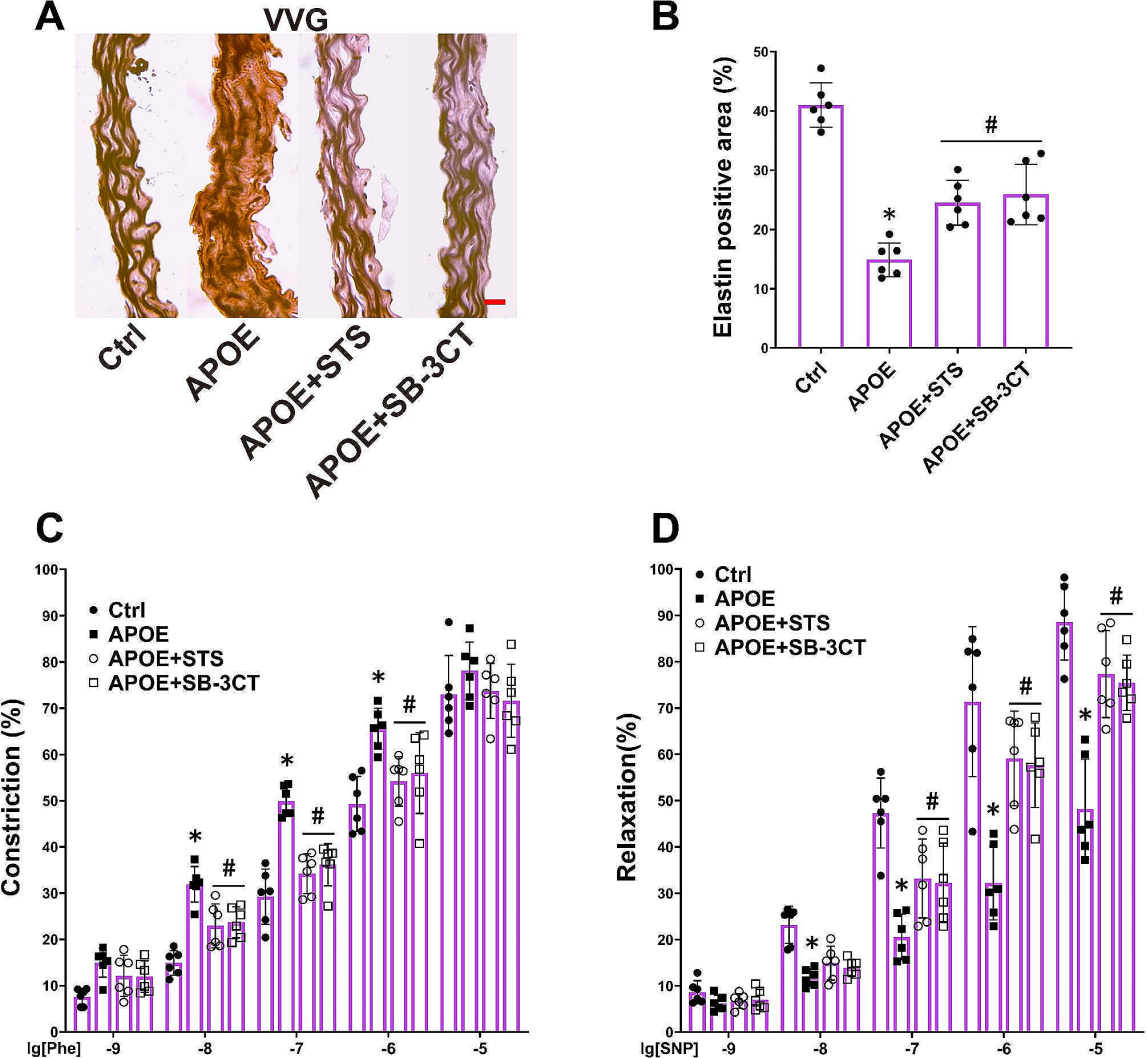
STS protects elastic fibers and vascular relaxation by inhibiting MMP2/9 in the vascular wall of ApoE-/- mice. (**A-B**): Representative VVG staining images (400×) and summarized data show the elastic fiber positive area in aortas (Data represent means ± SD, one-way ANOVA); (**C-D**): The summarized data of vascular response to Phe and SNP was determined in aorta rings (Data represent means ± SD, two-way ANOVA). *P < 0.05 vs. Control (Ctrl); #*P* < 0.05 vs. APOE-/- (*n* = 6, scale bar = 20 μm)

In order to investigate the potential mechanisms underlying STS-mediated elastic fiber protection, we employed the MMP2/9 inhibitor SB-3CT for intervention in ApoE-/- mice. We observed that SB-3CT inhibited elastic fibers damage in ApoE-/- mice (Fig. 3A and B) and reinstated the relaxant function and reduced excessive contractile function of the aorta in these mice (Fig. 3C and D). STS exhibited comparable effects to SB-3CT (Fig. 3A-D).

These findings suggest that STS maintains vascular relaxant function by suppressing the MMP2/9 pathway in a hyperlipidemic environment.

### STS protects elastic fiber and vascular relaxation of ApoE-/- mice by suppressing NLRP3-MMP2/9 pathway

**Fig. 4:**
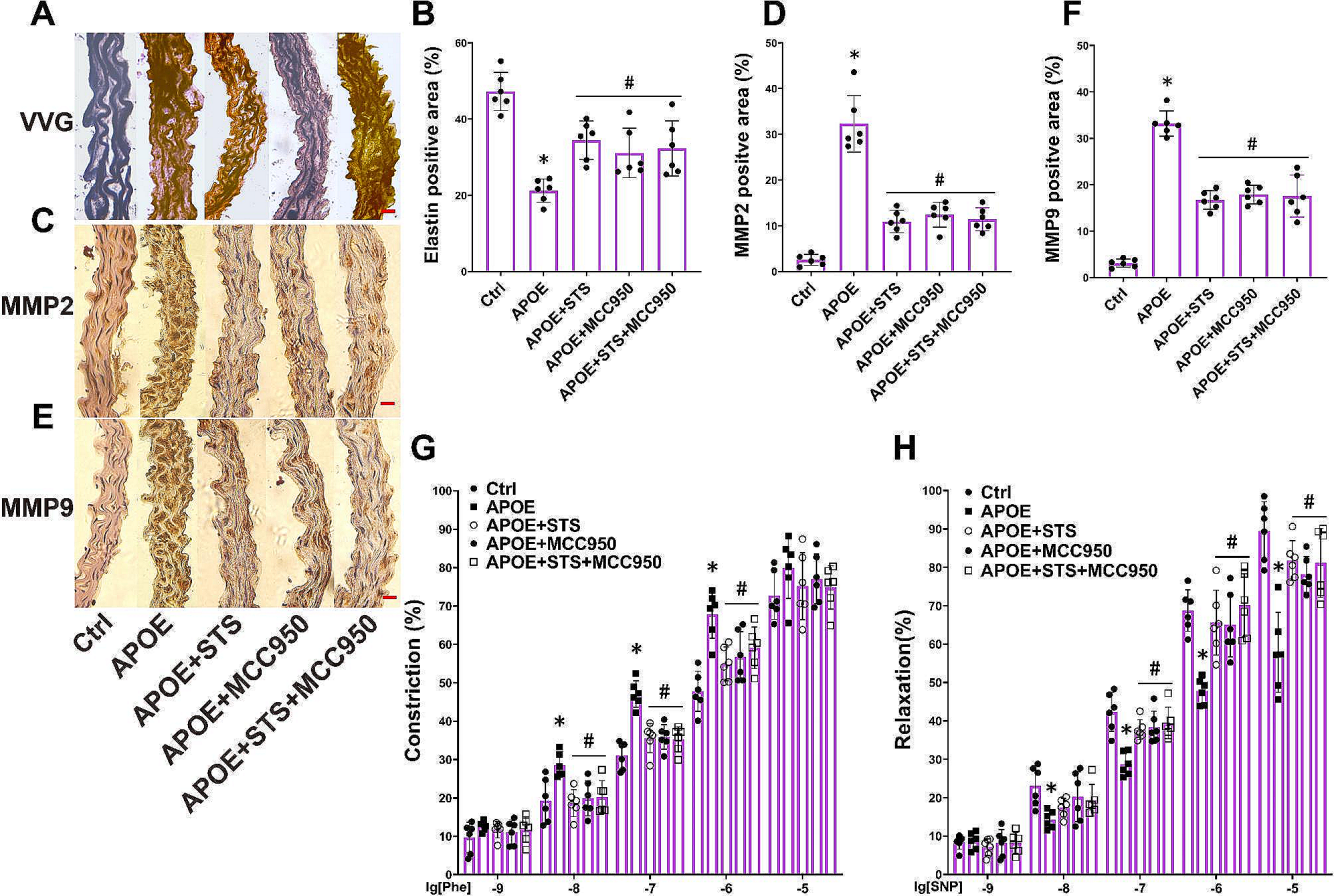
STS protects elastic fiber and vascular relaxation of ApoE-/- mice by suppressing NLRP3-MMP2/9 pathway. (**A-B**): Representative VVG staining images (400×) and summarized data show the elastic fiber positive area in aortas (Data represent means ± SD, one-way ANOVA); (**C-F**): Representative IHC images (400×) and summarized data show the MMP2 and MMP9 positive area in aortas (Data represent means ± SD, one-way ANOVA); (**G-H**): The summarized data of vascular response to Phe and SNP was determined in aorta rings (Data represent means ± SD, two-way ANOVA). *P < 0.05 vs. Control (Ctrl); #*P* < 0.05 vs. APOE-/- (*n* = 6, scale bar = 20 μm)

To explore the critical role of NLRP3 inflammasome in hyperlipidemia-induced relaxant function loss, we inhibited NLRP3 in ApoE-/- mice through MCC950 treating. We observed that MCC950 restored damaged elastic fibers in the aortic wall of ApoE-/- mice (Fig. 4A and B), reduced the expression levels of MMP2 and MMP9 (Fig. 4C-F), decreased the excessive contractile function, and ameliorated relaxant function of the aorta (Fig. 4G-H). STS exhibited similar effects to MCC950, and co-administration of STS and MCC950 did not yield a more pronounced pharmacological effect (Fig. 4A-H).

These results indicate that STS protects vascular elastic fiber and relaxation function in ApoE-/- mice by inhibiting the NLRP3-MMP2/9 pathway.

### STS protects elastic fiber and vascular relaxation of ApoE-/- mice by suppressing SYK-NLRP3 pathway

**Fig. 5:**
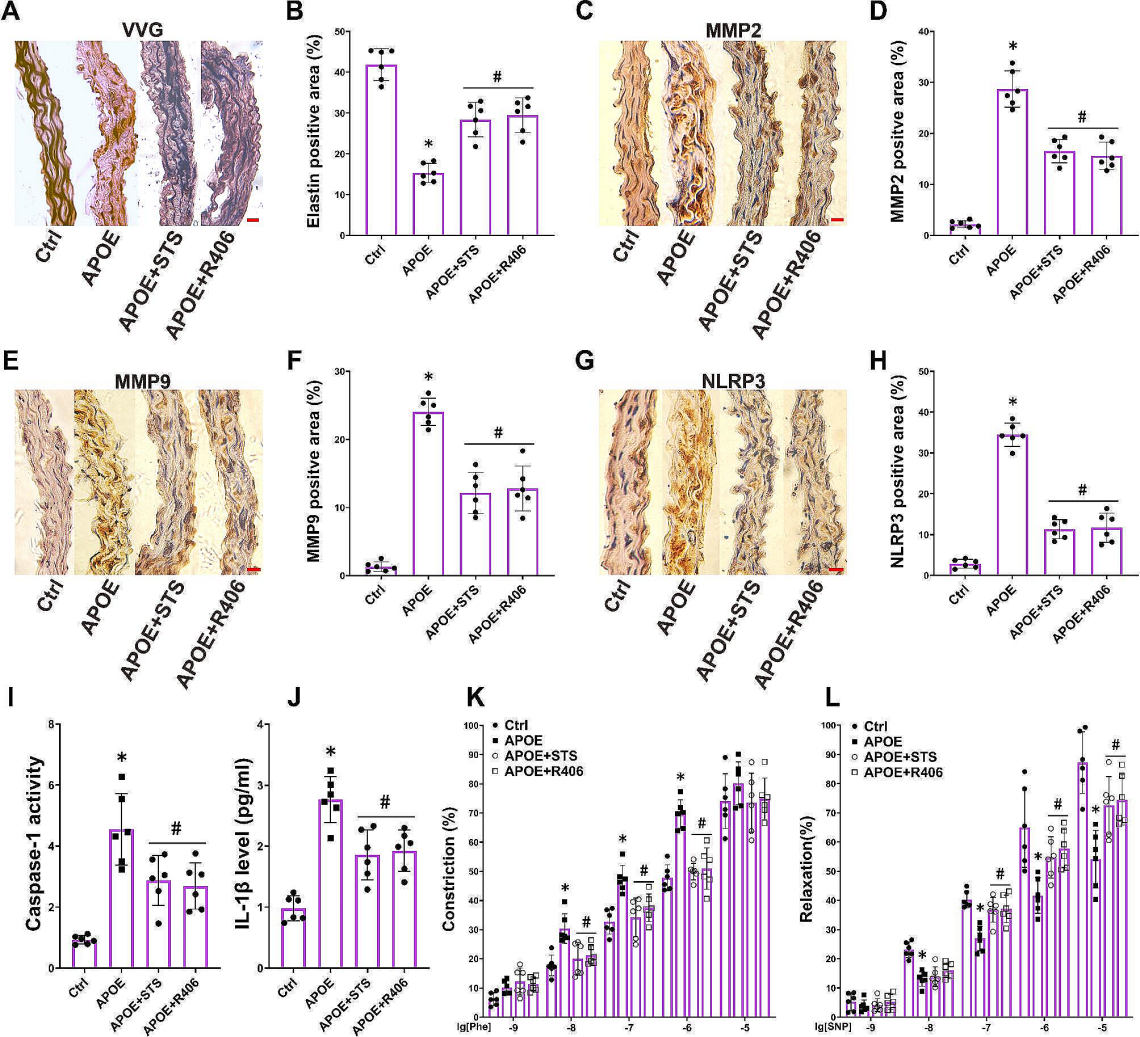
STS protects elastic fiber and vascular relaxation of ApoE-/- mice by suppressing SYK-NLRP3 pathway. (**A-B**): Representative VVG staining images (400×) and summarized data show the elastic fiber positive area in aortas (Data represent means ± SD, one-way ANOVA); (**C-H**): Representative IHC images (400×) and summarized data show the MMP2, MMP9 and NLRP3 positive area in aortas (Data represent means ± SD, one-way ANOVA); (**I-J**): The summarized data show the activity of caspase-1 and the levels of IL-1β in aorta homogenate (Data represent means ± SD, one-way ANOVA); (**K-L**): The summarized data of vascular response to Phe and SNP was determined in aorta rings (Data represent means ± SD, two-way ANOVA). *P < 0.05 vs. Control (Ctrl); #*P* < 0.05 vs. APOE-/- (*n* = 6, scale bar = 20 μm)

In order to investigate whether the protective effect of STS on vascular relaxant function in ApoE-/- mice is associated with the SYK pathway, the SYK-specific inhibitor R406 was used to block the SYK pathway. We found that R406 restored damaged elastic fibers in the aortic wall of ApoE-/- mice (Fig. 5A and B), reduced the expression levels of MMP2 and MMP9 (Fig. 5C-F), inhibited the positive percent of NLRP3, the levels of active caspase-1 and IL-1β (Fig. 5G-J), and attenuated the contractile function while reinstating the relaxant function of the aortas (Fig. 5K-L). STS mimicked the effects of R406 (Fig. 5A-L).

These results indicate that STS inhibits NLRP3 inflammasome activation and MMP2/9 expression, protects vascular relaxant function in ApoE-/- mice by inhibiting the SYK pathway.

## Discussion

The present study elucidates that STS maintains elastic fibers and protects vascular relaxation by inhibiting the SYK-NLRP3 inflammasome-MMP2/9 pathway. The findings of this study may offer a novel mechanism for hyperlipidemia-induced vascular relaxation damage, providing additional evidence for the vascular protective effects of STS.

Tanshinone IIA has been confirmed in several studies to exert protective effects on endothelial function and inhibit VSMC proliferation and migration by modulating oxidative stress, inflammation signaling pathways [17, 25]. However, the specific impact of tanshinone IIA and its sulfonated derivative (STS) on vascular elastic function has not been thoroughly investigated, especially in the pathological context of hyperlipidemia, where relevant research is notably lacking. Some studies suggest that tanshinone IIA preserves endothelial nitric oxide (NO) synthesis capacity, thereby protecting vascular relaxation function [26, 27]. Additionally, other research indicates that tanshinone IIA inhibits vascular calcification, fibrosis [28, 29]. These conclusions may suggest that tanshinone IIA alleviates vascular stiffness. Nevertheless, the applicability of these findings in a high-fat environment remains uncertain.

Our study unveils the novel pharmacological effects of STS in protecting vascular relaxation function. STS appears to maintain vascular relaxant function by safeguarding the integrity of vascular elastic fibers. Our research reveals that hyperlipidemia induces an upregulation of MMP2/9 levels in the vascular wall, leading to the loss of vascular elastic fibers and subsequent dysfunction in vascular relaxant function. STS, by protecting elastic fibers, sustains vascular relaxation. Moreover, our study suggests that the mechanism by which STS protects elastin may be associated with the inhibition of NLRP3 inflammasome activation.

The activation of the NLRP3 inflammasome is considered to be associated with the upregulation of MMP levels. In pulmonary fibrosis research, the activation of Nuclear Factor Kappa B (NF-κB)/NLRP3 signaling induces the upregulation of MMP2/9 expression [30]. Conversely, in the progression of chronic kidney disease, the knockout of NLRP3 is accompanied by the downregulation of MMP9 [31]. Therefore, the relationship between NLRP3 and MMPs may exhibit diversity and tissue specificity. Our study demonstrates that in the aortic wall of ApoE-/- mice, the activation of the NLRP3 inflammasome induces an upregulation of MMP2/9 levels. This conclusion aligns with previous studies, such as the inhibition of NLRP3 inflammasome, coincident with the downregulation of MMP2 expression in the lesion site of abdominal aortic aneurysm (AAA), and the prevention of elastin degradation [32]. Additionally, the inhibition of NLRP3 inflammasome activation, downregulation of MMP2/9 induced by high glucose environment, suppression of VSMC migration, and attenuation of neointima formation have been reported [13]. Furthermore, ox-LDL-induced toll-like receptor 4 (TLR4)/SYK/NLRP3 pathway upregulates MMP3/9 levels, inducing vascular senescence-related damage [33]. The NLRP3/MMP9 pathway induces VSMC apoptosis and elastic fiber degradation, contributing to the progression of intracranial aneurysms [14]. Thus, the activation of NLRP3 inflammasome is highly likely to be positively correlated with the upregulation of MMP2/9 and potentially other MMP types. In the progression of vascular diseases, the NLRP3 inflammasome-dependent excessive expression of MMPs is likely a key pathogenic factor contributing to vascular elastic dysfunction.

Previous research findings suggest that inhibiting the NLRP3/caspase-1 pathway in AAA models may alleviate elastin degradation at the aneurysmal site [24]. Similarly, in aortic aneurysm and dissection models, suppressing the expression of NLRP3 inflammasome components while simultaneously enhancing elastin protein expression has been observed [34]. Correspondingly, in a neointima hyperplasia model, activation of the NFκB-NLRP3 inflammasome pathway coincides with increased elastin fragmentation [35]. These conclusions are further evident in studies focusing on vascular elastic function. Inhibition of NLRP3 inflammasome activity in aortic endothelial cells restores vascular relaxation function impaired by cigarette smoke exposure [36]. Similarly, the alleviation of diabetic vascular senescence and protection of vascular relaxation function is observed upon NLRP3 knockout [21, 37]. Therefore, the NLRP3 inflammasome is highly likely to be a key mechanism underlying the impairment of vascular relaxation function. Our results corroborate this inference, demonstrating that inhibiting NLRP3 effectively restores impaired relaxation function in the aortas of ApoE-/- mice, reducing their sensitivity to vasoconstrictive stimuli. We connect the NLRP3-dependent upregulation of MMP2/9 levels to the damage of elastin fibers, resulting in the loss of vascular relaxation function. Furthermore, we investigate how STS mitigates vascular tension under hyperlipidemic conditions by inhibiting MMP2/9 and protecting elastin.

With our conclusions comes the crucial questions of how activated NLRP3 inflammasomes induce the expression or enzymatic activity of MMP2/9. It has been suggested that NLRP3 inflammasome-induced MMP expression may involve pathways such as IL-1β, TGF-β, and others [31, 38]. However, these conclusions await validation in VSMCs, and the gap between NLRP3 inflammasome activation and MMPs overexpression awaits further research to fill. Additionally, the balance between MMPs activity and their inhibitory protein tissue inhibitors of metalloproteinases (TIMPs) is a key mechanism in many human diseases [39]. Our study conclusions reveal changes in the expression levels of MMP2/9, and the relationship between their activity levels and TIMPs is likely a potential key mechanism of elastin fiber damage. Our study conclusions may inspire further research on these issues. Furthermore, hyperlipidemia may induce NLRP3 inflammasome activation in immune cells and produce large amounts of IL-1β, leading to phenotype transformation of VSMCs and synthesis and secretion of MMPs stimulated by IL-1β produced after immune cell infiltration [11]. Our findings demonstrate that cells in the vascular wall, primarily composed of VSMCs, produce large amounts of activated NLRP3 inflammasomes and induce overexpression of MMPs. Therefore, our conclusions may only touch on one aspect of this research area, and our conclusions urgently need validation using primary VSMCs.

It is worth noting that our current study conclusions only observed the damage and loss of elastin in the vascular wall. The metabolic processes, synthesis, secretion, crosslinking, degradation, and regeneration of elastin are likely altered by the pathological environment of hyperlipidemia, which may be a potential cause of the observed elastin damage [40, 41]. Our conclusions may provide some insights into the new research field of the relationship between these metabolic processes of elastin and NLRP3 inflammasomes.

Tanshinone IIA’s inhibition of MMP2/9 levels to alleviate the formation of atherosclerotic plaques has been extensively documented [42]. Furthermore, tanshinone IIA, while inhibiting MMP2/9, is reported to elevate elastin levels [43]. Our results further delineate the impact of STS (the sulfonate derivative of tanshinone IIA) in maintaining the integrity of elastic fiber and protecting vascular relaxation function against hyperlipidemic damage by suppressing the elevated expression of MMP2/9 mediated by the NLRP3 inflammasome. This study discovered that STS alleviates elevated left ventricular diastolic pressure in APOE-/- mice. We speculate that STS may alleviate left ventricular afterload under hyperlipidemic conditions. Arterial wall resistance is one of the main components of left ventricular afterload. We may indirectly demonstrate that STS partially alleviates arterial elastic damage induced by hyperlipidemia. This conclusion is partially consistent with previous research findings on STS alleviating pulmonary arterial hypertension [44, 45]. Although these data may suggest this conclusion, we need to cautiously propose that LVBP may be influenced by hemodynamics, endocrine status, renal function, and other factors in the pathological environment of hyperlipidemia. Despite using ex vivo vascular tissue devoid of various neural and humoral regulators to avoid interference from in vivo neurohumoral regulation on vascular wall tension, the results from these ex vivo tissues may still be influenced by neural elements on the adventitia that respond to changes in the ex vivo environment affecting vascular wall tension. Therefore, this conclusion awaits further integrated validation using more precise research methods, such as pulse wave velocity, etc.

Researches indicate that STS and its analogs possess pharmacological properties in inhibiting the NLRP3 inflammasome [46]. The current research findings suggest that the NLRP3 inflammasome is likely a critical target through which STS safeguards the vascular elasticity ability. However, the upstream mechanism through which STS inhibits the NLRP3 inflammasome remains intricate, particularly concerning its protective effects on vascular function. Further investigation into the molecular mechanisms is warranted. Our results propose that STS suppresses NLRP3 inflammasome activation by inhibiting the phosphorylation of SYK, consequently repressing MMP2/9 expression, preserving elastin, and maintaining vascular vasodilatation. Previous studies have suggested that tanshinone I inhibits SYK [47]. However, there is currently no reported evidence regarding whether STS possesses such characteristics. Our findings reveal that STS possesses the ability to inhibit SYK-mediated NLRP3 inflammasome activation, thereby enriching the understanding of the mechanisms by which STS inhibits the NLRP3 inflammasome. SYK controls both pro-IL-1β synthesis and NLRP3 activation in response to fungal infection [48]. Our previous studies have shown that the overactivation of the SYK-NLRP3 inflammasome pathway induces VSMC proliferation and migration in response to high-glucose environments [13]. However, whether this pathway is activated in the pathological environment of hyperlipidemia and its effects on vascular function remain to be investigated. Hyperlipidemia, SYK, and NLRP3 inflammasome are independently critical factors in various vascular injuries, but the relationship among them lacks relevant research at present. Our conclusion provides a new insight for research in this area. This novel mechanism of our study provides additional insights into the vascular protective effects associated with the anti-inflammatory properties of STS, potentially offering further foundational research evidence for its clinical application.

## Conclusion

In a hyperlipidemic environment, our study elucidated that SYK phosphorylation activates the NLRP3 inflammasome, leading to elevated MMP2/9 expression, which damages vascular wall elastic fibers and causes dysfunction in vascular relaxation. By inhibiting SYK phosphorylation-mediated NLRP3 pathway, STS suppresses MMP2/9, protects elastic fibers, and preserves vascular relaxation function in the ApoE-/- mice. Our research reveals a novel mechanism by which hyperlipidemia impairs vascular relaxation function, expands the cardiovascular protective effects of STS, and provides new foundational research evidence for its clinical application.

### Electronic supplementary material

Below is the link to the electronic supplementary material.

Supplementary Material 1

Supplementary Material 2

## Data Availability

The datasets used and analyzed in the current study are available from the corresponding author based on reasonable request.

## Abbreviations

STS: Sodium Tanshinone IIA Sulfonate
NLRP3: NOD-like receptor family pyrin domain-containing 3
ApoE-/- mice: ApoE-knockout mice
SYK: Spleen tyrosine kinase
ECs: Endothelial cells
VSMCs: Vascular smooth muscle cells
MMP2/9: Matrix metalloproteinase 2/9
TC: Total cholesterol
TG: Triglyceride
VVG: Verhoeff Van Gieson staining
LVBP: Left ventricular blood pressure
WT: Wild type mice
