# Supplementary material for: Sodium tanshinone IIA sulfonate protects vascular relaxation in ApoE-knockout mice by inhibiting the SYK-NLRP3 inflammasome-MMP2/9 pathway
Source: BMC Cardiovasc Disord. 2024 Jul 12;24:354. doi: 10.1186/s12872-024-03990-0 (PMC11241843; doi:10.1186/s12872-024-03990-0)
Supplement: Supplementary file 1 — Supplementary Material 1 [file 12872_2024_3990_MOESM1_ESM.pdf]

Sodium Tanshinone IIA Sulfonate Protects Vascular Relaxation in ApoE-Knockout Mice by Inhibiting the SYK-NLRP3 Inflammasome-MMP2/9 Pathway

Hai-Hua Liu[1], Wei Wei[1, 2, 3*], Fei-Fei Wu[1], Lu Cao[1], Bing-Jie Yang[4], Jia-Ning Fu[4], Jing-Xia Li[5], Xin-Yue Liang[6], Hao-Yu Dong[1], Yan-Yan Heng[7], Peng-Fei Zhang[7]

[1] Department of Endocrinology, Heping Hospital Affiliated to Changzhi Medical College, No.110, Yanan Road South, Changzhi, Shanxi, China, 046000.

[2] Department of Pharmacology, Changzhi Medical College, No.161, Jiefang East Street, Changzhi, Shanxi, China, 046000.

[3] Department of Clinical Center Laboratory, Heping Hospital Affiliated to Changzhi Medical College, No.110, Yan'an South Road, Changzhi, Shanxi, China, 046000.

[4] Department of Stomatology, Changzhi Medical College, No.161, Jiefang East Street, Changzhi, Shanxi, China, 046000.

[5] Department of Anesthesia, Changzhi Medical College, No.161, Jiefang East Street, Changzhi, Shanxi, China, 046000.

[6] Department of Medical Imageology, Changzhi Medical College, No.161, Jiefang East Street, Changzhi, Shanxi, China, 046000.

[7] Department of Nephrology Heping Hospital Affiliated to Changzhi Medical College, No.110, Yanan Road South, Changzhi, Shanxi, China, 046000.

Correspondence: Wei Wei, Ph.D., Department of Pharmacology, Changzhi Medical College, No.161, Jiefang East Street, Changzhi, Shanxi, China, 046000; Department of Endocrinology, Heping Hospital Affiliated to Changzhi Medical College, No.110,

Yan'an South Road, Changzhi, Shanxi, China, 046000; Department of Clinical Center Laboratory, Heping Hospital Affiliated to Changzhi Medical College, No.110, Yan'an South Road, Changzhi, Shanxi, China, 046000. Telephone: +86-0355-3128172; E-mail: jaywei@czmc.edu.cn.

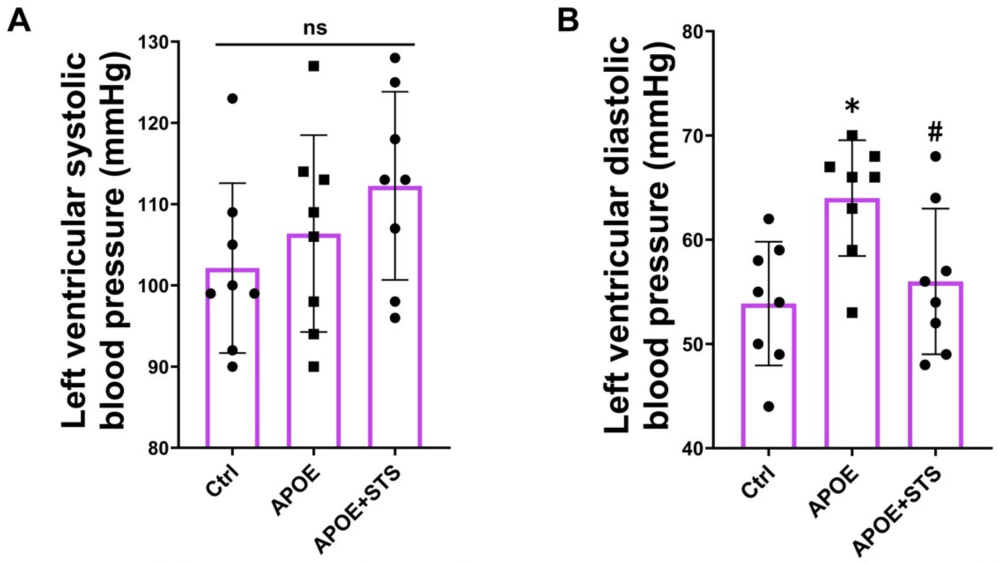

Supplemental figure 1. Left ventricular blood pressure (LVBP). A: The summarized data show Left ventricular systolic blood pressure; B: The summarized data show Left ventricular diastolic blood pressure. *P<0.05 vs. Control (Ctrl); #P<0.05 vs. APOE-/- (n=6, Data represent means ± SD, one-way ANOVA).

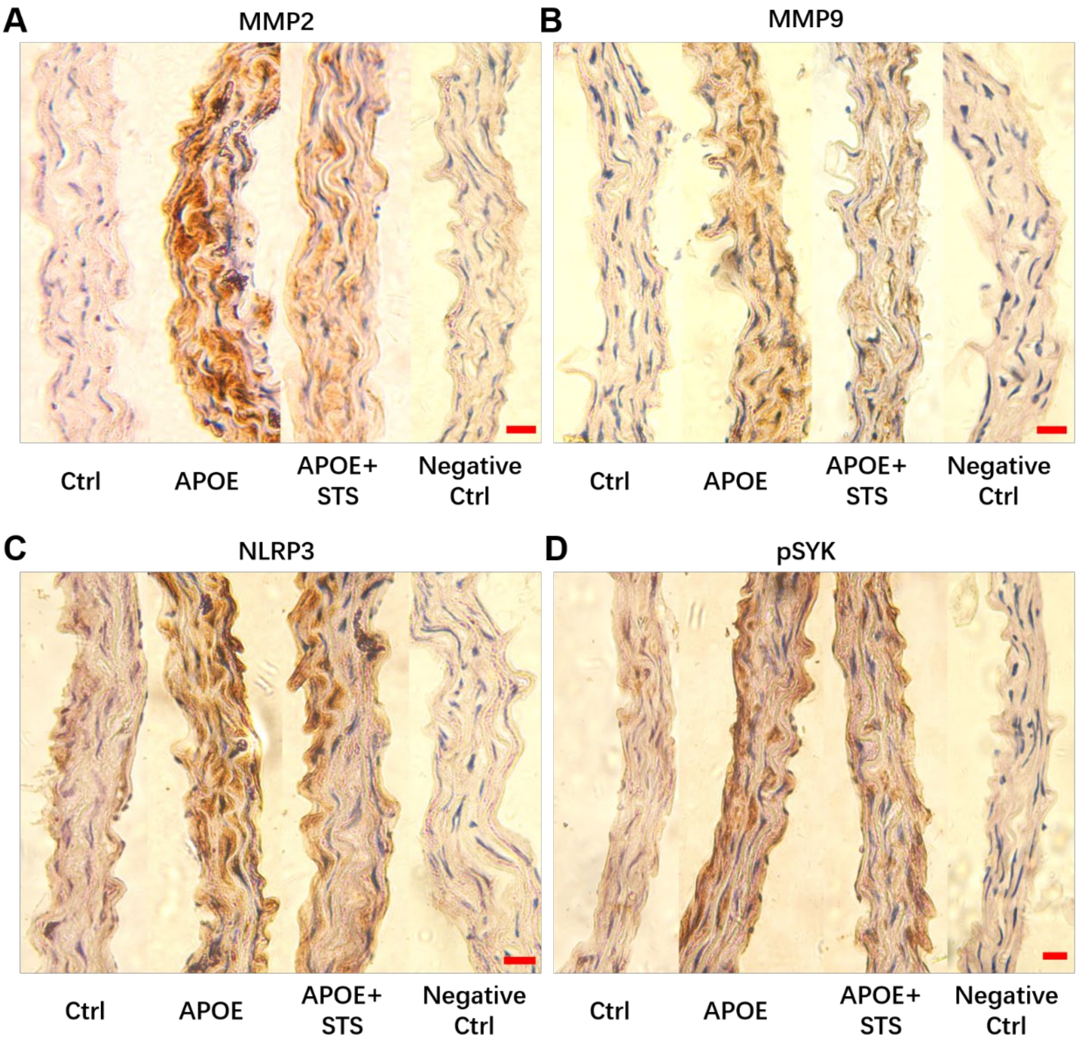

Supplemental figure 2. The non-specific binding control of IHC staining for (A) MMP2, (B) MMP9, (C) NLRP3 and (D) pSYK.

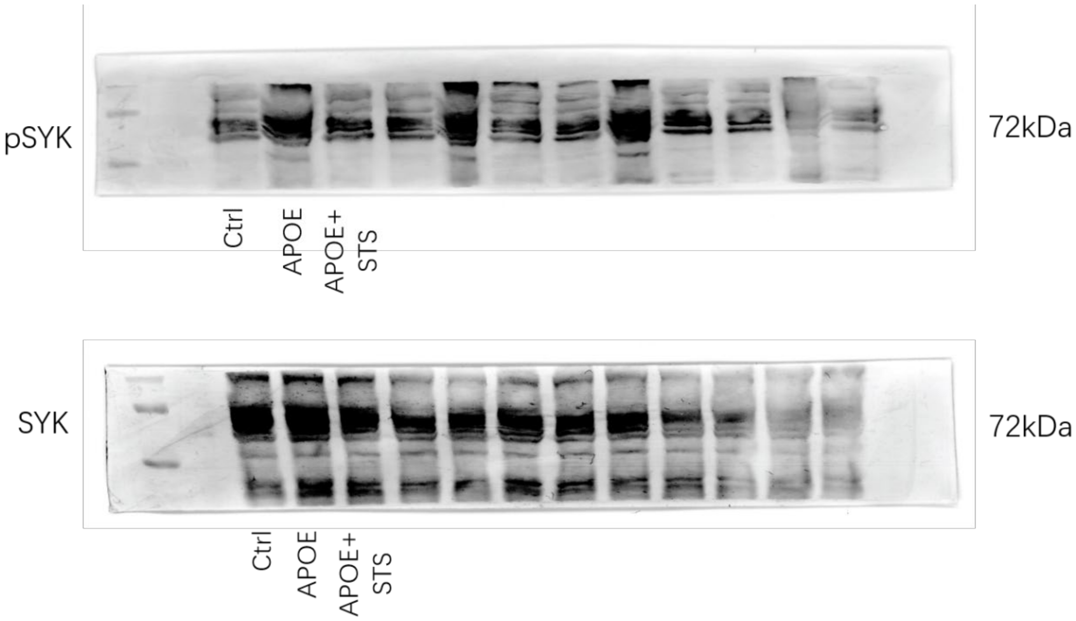

Supplemental figure 3. The full-length blots of pSYK and SYK.
